# Relationships between Global DNA Methylation in Circulating White Blood Cells and Breast Cancer Risk Factors

**DOI:** 10.1155/2017/2705860

**Published:** 2017-04-06

**Authors:** Nayha Chopra-Tandon, Haotian Wu, Kathleen F. Arcaro, Susan R. Sturgeon

**Affiliations:** ^1^Department of Biostatistics and Epidemiology, School of Public Health and Health Sciences, University of Massachusetts, Amherst, MA, USA; ^2^Department of Environmental Health Sciences, School of Public Health and Health Sciences, University of Massachusetts, Amherst, MA, USA; ^3^Department of Veterinary and Animal Science, College of Natural Sciences, University of Massachusetts, Amherst, MA, USA

## Abstract

It is not yet clear whether white blood cell DNA global methylation is associated with breast cancer risk. In this review we examine the relationships between multiple breast cancer risk factors and three markers of global DNA methylation:* LINE-1*, 5-mdC, and* Alu*. A literature search was conducted using Pubmed up to April 1, 2016, using combinations of relevant outcomes such as “WBC methylation,” “blood methylation,” “blood* LINE-1* methylation,” and a comprehensive list of known and suspected breast cancer risk factors. Overall, the vast majority of reports in the literature have focused on* LINE-1*. There was reasonably consistent evidence across the studies examined that males have higher levels of* LINE-1* methylation in WBC DNA than females. None of the other demographic, lifestyle, dietary, or health condition risk factors were consistently associated with* LINE-1* DNA methylation across studies. With the possible exception of sex, there was also little evidence that the wide range of breast cancer risk factors we examined were associated with either of the other two global DNA methylation markers: 5-mdC and* Alu*. One possible implication of the observed lack of association between global WBC DNA methylation and known breast cancer risk factors is that the association between global WBC DNA methylation and breast cancer, if it exists, is due to a disease effect.

## 1. Introduction

A CpG site, a cytosine followed by a guanine, has the potential to be methylated, and measuring 5-methyl-2′ deoxycytidine (5-mdC) content across the genome by liquid chromatography/mass spectrometry (LC/MS) can provide an overall measure of genome-wide DNA methylation levels. Repetitive sequences of the genome such as* LINE-1* and* Alu* contain up to half of all DNA methylation in the genome [1]. Thus, measuring DNA methylation levels in* LINE-1* or* Alu* repetitive elements by pyrosequencing or Methyl Light is often used as a surrogate higher-throughput approach to assess genome-wide methylation [2]. Genome instability has been associated with DNA hypomethylation and such global loss of methylation is common in breast tumor tissue [3–7].

There is some evidence that peripheral white blood cell (WBC) DNA contains epigenetic information that can be used to assess an individual's risk of breast cancer. In a case-control study of breast cancer of 179 cases and 180 controls, Choi and colleagues observed a nearly threefold increase in risk among women in the lowest tertile of total 5-mdC levels in WBC DNA compared to women in the highest tertile [1]. In the NIEHS sister case-cohort study of 294 cases and 646 noncases in which the mean time between blood collection and breast cancer diagnosis was 15 months [8],* LINE-1* methylation percentage in WBC DNA was also inversely associated with the risk of breast cancer, with a nearly twofold increased risk observed among women in the lowest quartile compared with those in the highest quartile. However, Brennan and colleagues reported no association between* LINE-1* WBC methylation and breast cancer risk in three prospective nested case-control studies [9]. Several other case-control studies, ranging in the number of breast cancer cases from 19 to 1064, found no association between* LINE-1* methylation and breast cancer risk [1, 10–12] or between* Alu* methylation and breast cancer risk [12, 13].

The LUminometric Methylation Assay (LUMA) measures levels of 5-mdC in a specific CmCGG motif which is found both in promoter regions of the genome and in repetitive elements [11]. Interestingly, one case-control study reported a twofold* increase* in risk of breast cancer among women with higher 5-mdC content compared to those with lower levels measured by LUMA. Another study reported no association [14] and a third study reported a strong inverse association between increasing tertiles of LUMA methylation and breast cancer risk [15].

It is not yet clear whether WBC DNA global methylation is associated with breast cancer risk [16]. If there is an association, one possible explanation is that the association represents environmental and lifestyle determinants of breast cancer that influence both DNA methylation and breast cancer risk. An alternative possibility is that, in response to very early breast cancer, a new clone of circulating lymphocytes arises that alters white blood cell DNA methylation [17]. If WBC DNA methylation is a marker of exposure associated with breast cancer risk, rather than a marker of early disease, it is reasonable to expect that white blood cell DNA methylation patterns would be more likely to be correlated with hormonal and other established or suspected risk factors for breast cancer. Terry and colleagues [18] reviewed literature up to 2011 on the relation between WBC DNA methylation patterns and a number of cancer risk factors. As the literature has expanded substantially, we updated the review, focusing on four demographic factors (age, sex, race/ethnicity, and education), three lifestyle factors (alcohol, smoking, and physical activity), three dietary factors (BMI, vegetable intake, and fruit intake), and eight health history and reproductive factors (menopause status, fetal birth weight, family history of breast cancer, age at menarche, age at first birth, parity, hormone replacement therapy, and endogenous hormones) that have been associated with breast cancer [19]. We also included folate in our review because although results have been mixed for breast cancer, folate is plausibly linked to DNA methylation [20]. We examined the relationships between these breast cancer risk factors and three markers of global DNA methylation:* LINE-1*, 5-mdC, and* Alu*. Our review comprises literature published through April 2016 and includes over 30 new studies that were not included in the 2011 review [18].

## 2. Methods

A literature search was conducted using Pubmed up to April 1, 2016. Searches were performed using combinations of relevant outcomes such as “WBC methylation,” “blood methylation,” “blood* LINE-1* methylation,” and a comprehensive list of known and suspected breast cancer risk factors such as “diet,” “physical activity,” and “menopause.” Boolean operators “and” and “or” were used whenever appropriate. Titles and abstracts were screened to determine relevancy by three independent reviewers. Additionally, bibliographies of select reviews were screened to ensure capture of all relevant information and ideas. If relevancy could not be determined from the abstract, the full text was retrieved to ensure comprehensive capture.

A study was included if it was primary research, published in English, and contained relevant results on any risk factor and blood DNA methylation outcomes. Studies were included that had both men and women due to the limited number of studies performed only in women. Studies were only included if their data were based on populations of nondiseased individuals.

## 3. Results

Table 1 shows the number of studies reporting associations between global WBC DNA methylation and demographic, lifestyle, dietary, and reproductive factors for each of three markers (i.e.,* LINE-1*,* Alu*, and 5-mdC). Overall, the vast majority of reports in the literature have focused on* LINE-1*. For example, 21 studies examined the association between age and* LINE-1* but only four studies examined age and 5-mdC. There were ten or more studies that each examined the association between* LINE-1* and alcohol, smoking, body mass index, vegetables, and folate. Fewer studies were available for* Alu* and 5-mdC and for reproductive risk factors.

### 3.1. Demographic Factors

#### 3.1.1. Age

As shown in Table 2, twenty of the twenty-one studies examining* LINE-1* reported no significant association between age and* LINE-1* methylation levels [11, 21–38]. Only one study reported a significant association between increasing age and higher* LINE-1* methylation levels [29]. Three of the six studies examining* Alu* methylation found no significant association with age [38, 41, 42], while the other three studies reported a significant association between increasing age and decreasing* Alu* methylation [22, 39, 40]. Two of four studies examining 5-mdC levels did not find a significant association with age [1, 44], while two other studies reported a statistically significant association between increasing age and decreasing 5-mdC levels [39, 43]. In summary, of the 31 studies with data on age and estimates of global DNA methylation no relationship was reported for 25 of the studies, a significant inverse relationship was reported for five studies, and a positive relationship was found in only one study.

#### 3.1.2. Sex

As shown in Table 2, eleven of the seventeen studies reported statistically significant higher* LINE-1* levels in males than in females [25, 28–30, 32, 34–36, 42, 45, 46], while the other six studies found no statistically significant association between sex and* LINE-1* levels [23, 27, 33, 37, 38, 47]. Two studies found no significant association between sex and* Alu* methylation [38, 40] while two other studies found a significant association for higher* Alu* methylation in males than in females [42, 48]. One study showed a significant association for higher 5-mdC levels in males than in females [43]. In summary, of the 22 studies with data on sex and estimates of global DNA methylation no relationship was reported for eight of the studies, and significant inverse relationship was reported for fourteen studies.

#### 3.1.3. Race/Ethnicity

Five studies investigated the association between* LINE-1* methylation and race/ethnicity (Table 2). Non-Hispanic Blacks had a significantly lower* LINE-1* level compared to non-Hispanic Whites [36] in one study, whereas the reverse was observed in two studies [28, 34]. Two other studies showed no significant association between race/ethnicity and* LINE-1* levels [11, 37]. One study showed no significant association between race/ethnicity and 5-mdC levels [1]. In summary, of the six studies with data on race/ethnicity and estimates of global DNA methylation no relationship was reported for three of the studies, non-Hispanic Blacks had a significantly lower global DNA methylation compared to non-Hispanic Whites in one of the studies, and the inverse relationship was reported for two of the studies.

#### 3.1.4. Education

As shown in Table 2, all six studies examining* LINE-1* that included education as a risk factor reported no significant association between the levels of education attained and* LINE-1* levels [21, 27, 29, 34, 36]. None of the studies examining* Alu* methylation included education as a risk factor. Only one 5-mdC study included education and it reported no significant association between the levels of education attained and 5-mdC levels [1]. In summary, of the seven studies with data on education and estimates of global DNA methylation no relationship was reported for any of the seven studies.

### 3.2. Lifestyle Factors

#### 3.2.1. Physical Activity

Five studies have investigated the association between physical activity and* LINE-1* levels (Table 3). Four studies found no significant difference between physical activity and* LINE-1* levels [24, 34, 37, 50] whereas one study reported that higher physical activity was associated with higher DNA methylation levels [49]. No studies examined the association between physical activity and* Alu* or 5-mdC. In summary, of the five studies with data on physical activity and estimates of global DNA methylation no relationship was reported for four of the studies and a positive relationship was found in one study.

#### 3.2.2. Alcohol

As shown in Table 3, thirteen studies examined* LINE-1* methylation and alcohol consumption. None of the thirteen studies reported a significant relationship between alcohol and* LINE-1* levels [11, 21, 27–29, 32–34, 36–38, 47]. Three studies found no significant association between alcohol and* Alu* methylation [38, 40, 41]. Additionally, of the only study that examined 5-mdC and alcohol consumption, there was no significant association between alcohol and 5-mdC levels [1]. In summary, of the 17 studies with data on alcohol and estimates of global DNA methylation no relationship was reported for any of the 17 studies.

#### 3.2.3. Smoking

As shown in Table 3, sixteen studies examined the relationship between* LINE-1* and smoking and all but one of the studies reported no significant association between* LINE-1* level and smoking habits [11, 21, 26–30, 32–36, 38, 47]. All four studies examining* Alu* found no association between smoking and* Alu* levels [38, 40–42], and both studies involving 5-mdC found no significant association between smoking and 5-mdC levels [1, 44]. In summary, of the 22 studies with data on smoking and estimates of global DNA methylation no relationship was reported for 21 of the studies and a significant inverse relationship was reported for one study with smokers having lower DNA methylation.

### 3.3. Dietary Factors

#### 3.3.1. BMI

A total of thirteen studies have examined the relationship between BMI and* LINE-1* levels (Table 3). Twelve studies reported no relationship [21, 24, 26, 29–31, 33, 34, 36–38, 47] while one study found that a higher BMI was statistically significantly associated with a lower* LINE-1* level [29]. Two studies found no significant association between BMI and* Alu* methylation [38, 40] while one study found that a higher BMI was significantly associated with a lower* Alu* methylation level [41]. One study found no relationship between BMI and 5-mdC levels [1]. In summary, of the 17 studies with data on BMI and estimates of global DNA methylation no relationship was reported for 15 of the studies, and significant inverse relationship was reported for two studies.

#### 3.3.2. Vegetables

As shown in Table 3, eight studies conducted found no relationship between vegetable intake and* LINE-1* levels [21, 24, 27, 29, 30, 37, 47]. Two other studies showed a significant association between lower vegetable intake and lower* LINE-1* methylation [29, 46]. One study found a significant association between higher adherence to a Mediterranean diet and lower* LINE-1* levels [51]. In summary, of the 11 studies with data on vegetables and estimates of global DNA methylation no relationship was reported for eight of the studies, and significant positive relationship was reported for three studies.

#### 3.3.3. Fruit

Six studies investigated the relationship between fruit intake and global white blood cell DNA methylation levels (Table 3). Five studies found no significant association between levels of fruit intake and* LINE-1* levels [27, 29, 37, 47]. One study found that, in women, there was a significant association between lower fruit intake and lower* LINE-1* levels [21]. In summary, of the six studies with data on fruit and estimates of global DNA methylation no relationship was reported for five of the studies, and significant positive relationship was reported for one study in women.

#### 3.3.4. Folate

As shown in Table 3, ten studies reported no significant relationship between dietary folate intake and* LINE-1* levels [11, 26–29, 34, 36, 44, 47]. Two studies reported a statistically significant positive correlation between higher blood folate levels and higher* LINE-1* levels [37, 52]. However, in one of the same studies, folate intake from natural foods and total dietary folate equivalents were not found to be associated with higher* LINE-1* levels [37]. Another study reported that women with a folate deficiency had a statistically significantly lower* LINE-1* level [21]. In summary, of the 13 studies with data on folate and global DNA methylation estimates no relationship was reported for ten of the studies and a significant positive relationship was reported for three of the studies.

### 3.4. Health History and Reproductive Factors

#### 3.4.1. Menopause Status

As shown in Table 3, one study examined the relationship between menopausal status and* LINE-1* methylation and did not find a significant association [11]. Another study did not find a significant association between menopausal status and 5-mdC levels [1].

#### 3.4.2. Fetal Birthweight

A cross-sectional study investigated the relationship between fetal birthweight and* LINE-1* levels and found a significant association between low (<2500 g) or high (4000+ g) birthweight and lower* LINE-1* levels of the newborn [53] (Table 3).

#### 3.4.3. Family History of Breast Cancer

Four studies reported no relationship between family history of breast cancer and* LINE-1* levels [9, 11, 54, 55] (Table 3). Family history of breast cancer was unrelated to 5-mdC levels in another study [1]. However, one study did find a relationship between family history of breast cancer and lower* Alu* levels [55]. In summary, of the six studies with data on family history and estimates of global DNA methylation no relationship was reported for five of the studies, and significant inverse relationship was reported for one study.

#### 3.4.4. Age at Menarche

One study found no association between the age at menarche and 5-mdC level [1] (Table 3).

#### 3.4.5. Age at First Birth/Parity

As seen in Table 3, one study did not find a significant association between the age at first live birth or parity, and 5-mdC level [1].

#### 3.4.6. Endogenous Hormones/Hormone Use

All four studies found no statistically significant association between* LINE-1* levels and sex hormone levels [56, 57] or between LINE-1 levels and phase of the menstrual cycle [25] (Table 3). The only study that evaluated 5-mdC levels did not find a significant association between 5-mdC and hormone use [1].

## 4. Discussion

There was reasonably consistent evidence across studies that males have higher levels of global methylation in WBC DNA than females. There was little evidence across studies that age was associated with global methylation in WBC DNA but the populations studied were generally restricted to older adults. Age has been reported to be associated with WBC DNA* LINE-1* methylation in a study that evaluated epigenetic changes throughout the lifetime of monozygotic twins [39]. None of the other demographic, lifestyle, dietary, or other risk factors were consistently associated with global WBC DNA methylation.

There are several factors that warrant consideration in interpreting the existing data on the associations between WBC DNA methylation and breast cancer risk factors. Nearly all the published studies used a composite of DNA from different subtypes of WBCs. As previously noted by others [18, 58], DNA methylation can vary by WBC subtype and the distribution of WBC subtypes varies among individuals, which could possibly obscure associations. In addition, the type and method of assessing WBC DNA methylation differed across studies, which could potentially contribute to variation in results across studies. Another possible explanation for the general lack of association with breast cancer risk factors is that the assays used may not be optimal. However, the fact that global WBC DNA methylation levels appear to be slightly lower among women than men when measured by any of the three assays tends to suggest that laboratory measurement error is not the entire explanation. Finally, studies are generally cross-sectional in design [18], and for some of the risk factors examined the number of studies was quite limited. Overall, however, it seems unlikely that these considerations account for the consistently null findings observed.

### 4.1. Conclusion

In summary, with the exception of sex, there is very little evidence that the wide range of breast cancer risk factors we examined (demographic, lifestyle, dietary, and health conditions) were associated with global WBC DNA methylation markers including* LINE-1*, 5-mdC, and* Alu*. Although the possibility that global DNA methylation reflects a novel breast cancer risk factor cannot be ruled out on the basis of these findings, a plausible implication of the observed lack of association between global WBC DNA methylation and most known or suspected breast cancer risk factors is that the association between global WBC DNA methylation and breast cancer, if it exists, is due to a disease effect [16, 59].

## Figures and Tables

**Table 1:** Summary of number of reports by risk factor and global methylation measure.

Factors	Total # of reports	*LINE-1*	*Alu*	5-mdC
Demographic Factors				
Age	31	21	6	4
Sex	22	17	4	1
Race/ethnicity	6	5	0	1
Education	7	6	0	1
Lifestyle factors				
Physical activity	5	5	0	0
Alcohol	17	13	3	1
Smoking	22	16	4	2
Dietary factors				
BMI	17	13	3	1
Vegetables	11	11	0	0
Fruit	6	6	0	0
Folate	13	12	0	1
Reproductive factors				
Menopause status	2	1	0	1
Fetal birthweight	1	1	0	0
Family history of breast cancer	6	4	1	1
Age at menarche	1	0	0	1
Age at first birth	1	0	0	1
Parity	1	0	0	1
Hormones	4	3	0	1

**Table 2:** Study Findings for demographic factors.

Authors	Methylation Type	Measurement method	Study participants	Findings	Comments
Age					

Agodi et al., 2015 [21]	LINE-1	Pyrosequencing	177 women aged 13–50, Helsinki	No differences	

Bollati et al., 2009 [22]	LINE-1	Pyrosequencing	718 individuals aged 55–92 from the Boston Area Normative Aging Study	No differences	

Chalitchagorn et al., 2004 [23]	LINE-1	COBRA PCR	32 individuals ranging in age, Thailand	No differences	

Duggan et al., 2014 [24]	LINE-1	Pyrosequencing	300 overweight women aged 50–75 in the US	No differences	

El-Maarri et al., 2011 [25]	LINE-1	Pyrosequencing, SIRPH	500 individuals aged 18–64, Bonn, Germany	No differences	

Gomes et al., 2012 [26]	LINE-1	ELISA	126 individuals aged 60–88, Brazil	No differences	

Hou et al., 2010 [27]	LINE-1	Pyrosequencing	421 individuals aged 21–79 in Warsaw, Poland	No differences	Data was stratified by gender. Before stratification, association with age was significant

Hsiung et al., 2007 [28]	LINE-1	COBRA PCR	765 individuals aged 18–75, Greater Boston Metropolitan Area	No differences	Adjusted for sex, race, smoking, alcohol, HPV serology, dietary folate, MTHFR

Karami et al., 2015 [29]	LINE-1	Pyrosequencing	PLCO - 436 controls from individuals aged 55–74 in the US, ATBC - 575 controls from individuals aged 55–69 in Finland	PLCO: No differences ATBC: significant difference between age groups (*p* < 0.001)	ATBC, increased age associated with higher methylation levels. Age 53-54 has 78.34 LINE-1 methylation%, 55–59 has 78.42 LINE-1 methylation%, 60–64 has 78.68 LINE-1 methylation%, 65–69 has 79.34 LINE-1 methylation%, 70–76 has 79.60 LINE-1 methylation%

Liao et al., 2011 [30]	LINE-1	Pyrosequencing	654 individuals aged 20–79 from the Central and Eastern European Renal Cancer Study (CEERCC)	No differences	

Marques-Rocha et al., 2016 [31]	LINE-1	MS-HRM	156 individuals aged 19–27, Brazil	No differences	

Mirabello et al., 2010 [32]	LINE-1	Pyrosequencing	314 individuals aged 12–75+ from the NCI Clinical Genetics Branch Familial TGTC Study in the US	No differences	Adjusted for sex

Pearce et al., 2012 [33]	LINE-1	Pyrosequencing	228 individuals aged 49–51 from Newcastle, England	No differences	

Perng et al., 2014 [34]	LINE-1	Pyrosequencing	987 adults aged 45–84 from the Multi-Ethnic Study of Atherosclerosis (MESA), NY & LA	No differences	

Wilhelm et al., 2010 [35]	LINE-1	Pyrosequencing	465 individuals aged 25–74, from NH	No differences	

Xu et al., 2012 [11]	LINE-1	Pyrosequencing	1101 women aged 20–98, from The Long Island Breast Cancer Study Project	No differences	

Zhang et al., 2011 [36]	LINE-1	Pyrosequencing	161 individuals aged 45–75 from the North Texas Healthy Heart Study	No differences	

Zhang et al., 2012 [37]	LINE-1	Pyrosequencing	165 individuals aged 18–78 from the COMIR (Commuting Mode and Inflammatory Response) study, NY	No differences	

Zhu et al., 2012 [38]	LINE-1	Pyrosequencing	1465 individuals total from a combination of 5 individual studies across MA; Warsaw, Poland; Milan, Italy; Brescia, Italy; Trissino, Italy	No differences	

Bollati et al., 2009 [22]	Alu	Pyrosequencing	718 individuals aged 55–92 from the Boston Area Normative Aging Study	Significant differences (*p* = 0.012) between age groups	Increased age associated with an average 0.2 5-mdC percentage decrease

Fraga et al., 2005 [39]	Alu	Total 5-mdC content: HPCE Sequence specific: bisulfite sequencing	80 monozygotic twins aged 3–74, Spain	Significant differences (*p* < 0.05) between age groups	Youngest pairs of MZ twins epigenetically similar, whereas oldest pairs clearly distinct

Kim et al., 2010 [40]	Alu	Pyrosequencing	86 individuals aged 42–69, South Korea	Significant differences (*p* = 0.03) between age groups	Statistically significant inverse association with DNA methylation. Adjusted for age

Na et al., 2014 [41]	Alu	Pyrosequencing	244 women aged 20–51, Korea	No differences	

Rusiecki et al., 2008 [42]	Alu	Pyrosequencing	70 individuals aged 19–67 from Greenlandic Inuit, Greenland	No differences	

Zhu et al., 2012 [38]	Alu	Pyrosequencing	1465 individuals total from a combination of 5 individual studies across MA; Warsaw, Poland; Milan, Italy; Brescia, Italy; Trissino, Italy	No differences	

Choi et al., 2009 [1]	5-mdC	LC/ESI-MS/MS	180 women aged 35–75	No differences	

Fraga et al., 2005 [39]	5-mdC	Total 5-mdC content: HPCE Sequence specific: bisulfite sequencing	80 monozygotic twins aged 3–74, Spain	Significant differences (*p* < 0.05) between age groups	Youngest pairs of MZ twins epigenetically similar, whereas oldest pairs clearly distinct

Fuke et al., 2004 [43]	5-mdC	HPLC	76 individuals aged 4–94	Significant differences (*p* = 0.0002) between age groups	Increased age associated with decreased methylation levels. Age 4–14 has 4.018% metC/dC + metC, age 16–22 has 4.03%, age 25–41 has 3.977%, and age 51–94 has 3.948%

Moore et al., 2008 [44]	5-mdC	HPCE, HpaII digest, densitometry	397 individuals aged 20–81 from the Spanish Bladder Cancer Study, Spain	No differences	

Sex					

Andreotti et al., 2014 [45]	LINE-1	Pyrosequencing	676 individuals aged 55–74 from the Prostate, Lung, Colorectal, Ovarian Cancer Screening Trial (PLCO) in the US	Significant differences (*p* = 0.0004) between male and female	Males had 84.2% average LINE-1 methylation%, Females had 83.5% average LINE-1 methylation%

Cash et al., 2012 [46]	LINE-1	Pyrosequencing	528 individuals aged 25–74 from the Residents Registry of the Shanghai Municipal Government, China	Significant differences (*p* = 0.0004) between male and female	Males had 82.09 average LINE-1 methylation%, Females had 81.53% average LINE-1 methylation%

Chalitchagorn et al., 2004 [23]	LINE-1	COBRA PCR	32 individuals ranging in age, Thailand	No differences	

El-Maarri et al., 2011 [25]	LINE-1	Pyrosequencing, SIRPH	500 individuals aged 18–64, Bonn, Germany	Significant differences (*p* = 0.01) between male and female	Average gender difference 0.94%

Hou et al., 2010 [27]	LINE-1	Pyrosequencing	421 individuals aged 21–79 in Warsaw, Poland	No differences	

Hsiung et al., 2007 [28]	LINE-1	Cobra PCR	765 individuals aged 18–75, Greater Boston Metropolitan Area	Significant differences (*p* = 0.002) between “male” and “female”	Not given; adjusted for age, race, smoking, alcohol, HPV serology, dietary folate, MTHFR

Karami et al., 2015 [29]	LINE-1	Pyrosequencing	PLCO, 436 controls from individuals aged 55–74 in the US	PLCO, Significant differences (*p* < 0.0001) between male and female	Males had 77.15% average LINE-1 methylation%, females had 76.58% average LINE-1 methylation%

Liao et al., 2011 [30]	LINE-1	Pyrosequencing	654 individuals aged 20–79 from the Central and Eastern European Renal Cancer Study (CEERCC)	Significant differences (*p* = 0.0003) between male and female	Males had 81.97% average LINE-1 methylation%, females had 81.4% average LINE-1 methylation%

Mirabello et al., 2010 [32]	LINE-1	Pyrosequencing	314 individuals aged 12–75+ from the NCI Clinical Genetics Branch Familial TGTC Study in the US	Significant differences (*p* = 0.002) between male and female	Males had 79.6% average LINE-1 methylation%, females had 78.87% average LINE-1 methylation%. Adjusted for age

Pearce et al., 2012 [33]	LINE-1	Pyrosequencing	228 individuals aged 49–51 from Newcastle, England	No differences	

Perng et al., 2014 [34]	LINE-1	Pyrosequencing	987 adults aged 45–84 from the Multi-Ethnic Study of Atherosclerosis (MESA), NY & LA	Significant differences (*p* = 0.0001) between male and female	Males had 80.94% average LINE-1 methylation%, Females had 80.54% average LINE-1 methylation%

Rusiecki et al., 2008 [42]	LINE-1	Pyrosequencing	70 individuals aged 19–67 from Greenlandic Inuit, Greenland	Significant differences (*p* = 0.02) between male and female	Males had 79.05% average LINE-1 methylation%, Females had 77.73% average LINE-1 methylation%

Tajuddin et al., 2013 [47]	LINE-1	Pyrosequencing	892 individuals aged 20–81 from the Spanish Bladder Cancer/EPICURO study, Spain	No differences	Significant differences (*p* = 0.02) between male and female before Bonferroni correction

Wilhelm et al., 2010 [35]	LINE-1	Pyrosequencing	465 individuals aged 25–74, from NH	Significant differences (*p* = 0.04) between male and female	Not given

Zhang et al., 2011 [36]	LINE-1	Pyrosequencing	161 individuals aged 45–75 from the North Texas Healthy Heart Study	Significant differences (*p* = 0.0001) between male and female	Males had 75% average LINE-1 methylation%, females had 73.2% average LINE-1 methylation%

Zhang et al., 2012 [37]	LINE-1	Pyrosequencing	165 individuals aged 18–78 from the COMIR (Commuting Mode and Inflammatory Response) study, NY	No differences	

Zhu et al., 2012 [38]	LINE-1	Pyrosequencing	1465 individuals total from a combination of 5 individual studies across MA; Warsaw, Poland; Milan, Italy; Brescia, Italy; Trissino, Italy	No differences	

El-Maarri et al., 2007 [48]	Alu	SIRPH	192 individuals aged 18–43, Bonn, Germany	Significant differences (*p* < 0.0003) between male and female	Slightly higher methylation in males

Kim et al., 2010 [40]	Alu	Pyrosequencing	86 individuals aged 42–69, South Korea	No differences	Adjusted for age

Rusiecki et al., 2008 [42]	Alu	Pyrosequencing	70 individuals aged 19–67 from Greenlandic Inuit, Greenland	Significant differences (*p* = 0.0001) between male and female	Males had 25.35% average Alu methylation%, Females had 24.69% average Alu methylation%

Zhu et al., 2012 [38]	Alu	Pyrosequencing	1465 individuals total from a combination of 5 individual studies across MA; Warsaw, Poland; Milan, Italy; Brescia, Italy; Trissino, Italy	No differences	

Fuke et al., 2004 [43]	5-mdC	HPLC	76 individuals aged 4–94	Significant differences (*p* < 0.0067) between male and female	Males had metC/(dC + metC) = 4.01 ± 0.069, females had metC/(dC + metC) = 3.975 ± 0.067

Race/Ethnicity					

Hsiung et al., 2007 [28]	LINE-1	Cobra PCR	765 individuals aged 18–75, Greater Boston Metropolitan Area	Significant differences (*p* = 0.03) between “non-Caucasian” and “Caucasian”	Not provided; Adjusted for age, sex, smoking, alcohol, HPV serology, dietary folate, MTHFR

Perng et al., 2014 [34]	LINE-1	Pyrosequencing	987 adults aged 45–84 from the Multi-Ethnic Study of Atherosclerosis (MESA), NY & LA	Significant differences (*p* = 0.008) found between “Caucasian Whites”, “African-American Blacks”, and Hispanics	Caucasian Whites had 80.5% average LINE-1 methylation%, African-American Blacks had 80.84% average LINE-1 methylation%, Hispanics had 80.75% average LINE-1 methylation%

Xu et al., 2012 [11]	LINE-1	Pyrosequencing	1101 women aged 20–98, from The Long Island Breast Cancer Study Project	No differences	

Zhang et al., 2011 [36]	LINE-1	Pyrosequencing	161 individuals aged 45–75 from the North Texas Healthy Heart Study	Significant differences (*p* = 0.001) found between “non-Hispanic Whites”, “non-Hispanic Blacks”, and Hispanics	Non-Hispanic Whites had 75.3% average LINE-1 methylation%, non-Hispanic Blacks had 73.1% average LINE-1 methylation%, Hispanics had 74% average LINE-1 methylation%

Zhang et al., 2012 [37]	LINE-1	Pyrosequencing	165 individuals aged 18–78 from the COMIR (Commuting Mode and Inflammatory Response) study, NY	No differences	

Choi et al., 2009 [1]	5-mdC	LC/ESI-MS/MS	180 women aged 35–75	No differences	

Education					

Agodi et al., 2015 [21]	LINE-1	Pyrosequencing	177 women aged 13–50, Helsinki	No differences	

Hou et al., 2010 [27]	LINE-1	Pyrosequencing	421 individuals aged 21–79 in Warsaw, Poland	No differences	

Karami et al., 2015 [29]	LINE-1	Pyrosequencing	PLCO - 436 controls from individuals aged 55–74 in the US, ATBC - 575 controls from individuals aged 55–69 in Finland	PLCO -No differences ATBC -No differences	

Perng et al., 2014 [34]	LINE-1	Pyrosequencing	987 adults aged 45–84 from the Multi-Ethnic Study of Atherosclerosis (MESA), NY & LA	No differences	

Zhang et al., 2011 [36]	LINE-1	Pyrosequencing	161 adults aged 45–75 from the North Texas Healthy Heart Study	No differences	

Choi et al., 2009 [1]	5-mdC	LC/ESI-MS/MS	180 women aged 35–75	No differences	

**Table 3:** Study findings for lifestyle, dietary, and reproductive factors.

Authors	Methylation Type	Measurement method	Study participants	Findings	Comments
Physical activity					

Duggan et al., 2014 [24]	LINE-1	Pyrosequencing	300 overweight women aged 50–75 in the US	No differences	

Perng et al., 2014 [34]	LINE-1	Pyrosequencing	987 adults aged 45–84 from the Multi-Ethnic Study of Atherosclerosis (MESA), NY & LA	No differences	

White et al., 2013 [49]	LINE-1	Pyrosequencing	647 non-Hispanic white women aged 35–74 from the NIH sister study, USA	Significant differences (*p* = 0.04) between “0” and “3” physical activity duration level above the median of physical activity	Physical activity levels of women greater than or equal to the median of physical activity at three time points (ages 5–12, 13–19 and currently) had higher global methylation compared to women with activity levels below the median for all three time periods (beta = .33, 95% CI: .01, 0.66)

Zhang et al., 2011 [50]	LINE-1	MethyLight	161 individuals aged 45–75 from the North Texas Healthy Heart Study	No differences	

Zhang et al., 2012 [37]	LINE-1	Pyrosequencing	165 individuals aged 18–78 from the COMIR (Commuting Mode and Inflammatory Response) study, NY	No differences	

Alcohol					

Agodi et al., 2015 [21]	LINE-1	Pyrosequencing	177 women aged 13–50, Helsinki	No differences	

Hou et al., 2010 [27]	LINE-1	Pyrosequencing	421 individuals aged 21–79 in Warsaw, Poland	No differences	

Hsiung et al., 2007 [28]	LINE-1	COBRA PCR	765 individuals aged 18–75, Greater Boston Metropolitan Area	No differences	Adjusted for age, sex, race, smoking, HPV serology, dietary folate, MTHFR

Karami et al., 2015 [29]	LINE-1	Pyrosequencing	PLCO - 436 controls from individuals aged 55–74 in the US, ATBC, 575 controls from individuals aged 55–69 in Finland	PLCO -No differences ATBC - No differences	

Mirabello et al., 2010 [32]	LINE-1	Pyrosequencing	314 individuals aged 12–75+ from the NCI Clinical Genetics Branch Familial TGTC Study in the US	No differences	

Pearce et al., 2012 [33]	LINE-1	Pyrosequencing	228 individuals aged 49–51 from Newcastle, England	No differences	

Tajuddin et al., 2013 [47]	LINE-1	Pyrosequencing	892 individuals aged 20–81 from the Spanish Bladder Cancer/EPICURO study, Spain	No differences	

Perng et al., 2014 [34]	LINE-1	Pyrosequencing	987 adults aged 45–84 from the Multi-Ethnic Study of Atherosclerosis (MESA), NY & LA	No differences	

Xu et al., 2012 [11]	LINE-1	Pyrosequencing	1101 women aged 20–98, from The Long Island Breast Cancer Study Project	No differences	

Zhang et al., 2011 [36]	LINE-1	Pyrosequencing	161 individuals aged 45–75 from the North Texas Healthy Heart Study	No differences	

Zhang et al., 2012 [37]	LINE-1	Pyrosequencing	165 individuals aged 18–78 from the COMIR (Commuting Mode and Inflammatory Response) study, NY	No differences	

Zhu et al., 2012 [38]	LINE-1	Pyrosequencing	1465 individuals total from a combination of 5 individual studies across MA; Warsaw, Poland; Milan, Italy; Brescia, Italy; Trissino, Italy	No differences	

Zhu et al., 2012 [38]	Au	Pyrosequencing	1465 individuals total from a combination of 5 individual studies across MA; Warsaw, Poland; Milan, Italy; Brescia, Italy; Trissino, Italy	No differences	

Kim et al., 2010 [40]	Alu	Pyrosequencing	86 individuals aged 42–69, South Korea	No differences	Adjusted for age

Na et al., 2014 [41]	Alu	Pyrosequencing	244 women aged 20–51, Korea	No differences	

Choi et al., 2009 [1]	5-mdC	LC/ESI-MS/MS	180 women aged 35–75	No differences	

Smoking					

Agodi et al., 2015 [21]	LINE-1	Pyrosequencing	177 women aged 13–50, Helsinki	No differences	

Andreotti et al., 2014 [45]	LINE-1	Pyrosequencing	676 individuals aged 55–74 from the Prostate, Lung, Colorectal, Ovarian Cancer Screening Trial (PLCO) in the US	No difference for females. Significant differences (*p* = 0.008) between “Never” and “Ever” smokers for males	“Never” smoked had 84% average LINE-1 methylation% and “Ever” smoked had 83.6% average LINE-1 methylation% for males

Gomes et al., 2012 [26]	LINE-1	ELISA	126 individuals aged 60–88, Brazil	No differences	

Hou et al., 2010 [27]	LINE-1	Pyrosequencing	421 individuals aged 21–79 in Warsaw, Poland	No differences	

Hsiung et al., 2007 [28]	LINE-1	COBRA PCR	765 individuals aged 18–75, Greater Boston Metropolitan Area	No differences	Adjusted for age, sex, race, alcohol, HPV serology, dietary folate, MTHFR

Karami et al., 2015 [29]	LINE-1	Pyrosequencing	PLCO - 436 controls from individuals aged 55–74 in the US	PLCO - No differences for females. Significant difference (*p* = 0.02) between smokers and nonsmokers for males	PLCO, males who had never smoked have an average 77.35% LINE-1 methylation%, and males who had ever smoked have an average 77.02% LINE-1 methylation%

Liao et al., 2011 [30]	LINE-1	Pyrosequencing	654 individuals aged 20–79 from the Central and Eastern European Renal Cancer Study (CEERCC)	No differences	

Mirabello et al., 2010 [32]	LINE-1	Pyrosequencing	314 individuals aged 12–75+ from the NCI Clinical Genetics Branch Familial TGTC Study in the US	No differences	

Pearce et al., 2012 [33]	LINE-1	Pyrosequencing	228 individuals aged 49–51 from Newcastle, England	No differences	

Perng et al., 2014 [34]	LINE-1	Pyrosequencing	987 adults aged 45–84 from the Multi-Ethnic Study of Atherosclerosis (MESA), NY & LA	No differences	

Tajuddin et al., 2013 [47]	LINE-1	Pyrosequencing	892 individuals aged 20–81 from the Spanish Bladder Cancer/EPICURO study, Spain	No differences	Adjusted for age, sex, region

Wilhelm et al., 2010 [35]	LINE-1	Pyrosequencing	465 individuals aged 25–74, from NH	No differences	

Xu et al., 2012 [11]	LINE-1	Pyrosequencing	1101 women aged 20–98, from The Long Island Breast Cancer Study Project	No differences	

Zhang et al., 2011 [36]	LINE-1	Pyrosequencing	161 individuals aged 45–75 from the North Texas Healthy Heart Study	No differences	

Zhu et al., 2012 [38]	LINE-1	Pyrosequencing	1465 individuals total from a combination of 5 individual studies across MA; Warsaw, Poland; Milan, Italy; Brescia, Italy; Trissino, Italy	No differences	

Kim et al., 2010 [40]	Alu	Pyrosequencing	86 individuals aged 42–69, South Korea	No differences	Adjusted for age

Na et al., 2014 [41]	Alu	Pyrosequencing	244 women aged 20–51, Korea	No differences	

Rusiecki et al., 2008 [42]	Alu	Pyrosequencing	70 individuals aged 19–67 from Greenlandic Inuit, Greenland	No differences	

Zhu et al., 2012 [38]	Alu	Pyrosequencing	1465 individuals total from a combination of 5 individual studies across MA; Warsaw, Poland; Milan, Italy; Brescia, Italy; Trissino, Italy	No differences	

Choi et al., 2009 [1]	5-mdC	LC/ESI-MS/MS	180 women aged 35–75	No differences	

Moore et al., 2008 [44]	5-mdC	HPCE, HpaII digest, densitometry	397 individuals aged 20–81 from the Spanish Bladder Cancer Study, Spain	No differences	

BMI					

Agodi et al., 2015 [21]	LINE-1	Pyrosequencing	177 women aged 13–50, Helsinki	No differences	

Duggan et al., 2014 [24]	LINE-1	Pyrosequencing	300 overweight women aged 50–75 in the US	No differences	

Gomes et al., 2012 [26]	LINE-1	ELISA	126 individuals aged 60–88, Brazil	No differences	

Karami et al., 2015 [29]	LINE-1	Pyrosequencing	PLCO, 436 controls from individuals aged 55–74 in the US, ATBC, 575 controls from individuals aged 55–69 in Finland	PLCO, no differences. ATBC, significant differences between 16.7–<25, 25–30, and 30–62.1	BMI 16.7–<25 had 79.00% average LINE-1 methylation%, BMI 25–30 had 78.73% average LINE-1 methylation%, and BMI 30–62.1 had 78.39% average LINE-1 methylation%

Liao et al., 2011 [30]	LINE-1	Pyrosequencing	654 individuals aged 20–79 from the Central and Eastern European Renal Cancer Study (CEERCC)	No differences	

Marques-Rocha, 2016 [31]	LINE-1	MS-HRM	156 individuals aged 19–27, Brazil	No differences	

Pearce et al., 2012 [33]	LINE-1	Pyrosequencing	228 individuals aged 49–51 from Newcastle, England	No differences	Adjusted for sex

Perng et al., 2014 [34]	LINE-1	Pyrosequencing	987 adults aged 45–84 from the Multi-Ethnic Study of Atherosclerosis (MESA), NY & LA	No differences	

Tajuddin et al., 2013 [47]	LINE-1	Pyrosequencing	892 individuals aged 20–81 from the Spanish Bladder Cancer/EPICURO study, Spain	No differences	

Zhang et al., 2011 [36]	LINE-1	Pyrosequencing	161 individuals aged 45–75 from the North Texas Healthy Heart Study	No differences	

Zhang et al., 2012 [37]	LINE-1	Pyrosequencing	165 individuals aged 18–78 from the COMIR (Commuting Mode and Inflammatory Response) study, NY	No differences	In unadjusted models, there was a statistically significant difference (*p* = 0.03)

Zhu et al., 2012 [38]	LINE-1	Pyrosequencing	1465 individuals total from a combination of 5 individual studies across MA; Warsaw, Poland; Milan, Italy; Brescia, Italy; Trissino, Italy	No differences	

Kim et al., 2010 [40]	Alu	Pyrosequencing	86 individuals aged 42–69, South Korea	No differences	Adjusted for age

Na et al., 2014 [41]	Alu	Pyrosequencing	244 women aged 20–51, Korea	Significant difference (*p* < 0.001) between normal weight, overweight, and obese groups	Normal weight had 26.28 Alu methylation%, overweight had 24.95 Alu methylation%, normal weight had 25.96 Alu methylation%

Zhu et al., 2012 [38]	Alu	Pyrosequencing	1465 individuals total from a combination of 5 individual studies across MA; Warsaw, Poland; Milan, Italy; Brescia, Italy; Trissino, Italy	No differences	

Choi et al., 2009 [1]	5-mdC	LC/ESI-MS/MS	180 women aged 35–75	No differences	

Vegetables					

Agodi et al., 2015 [21]	LINE-1	Pyrosequencing	177 women aged 13–50, Helsinki	No differences	

Cash et al., 2012 [46]	LINE-1	Pyrosequencing	528 individuals aged 25–74 from the Residents Registry of the Shanghai Municipal Government, China	Significant differences (*p* = 0.002) between “<4 times/week” and “≥4 times/week” intake of total cruciferous vegetables in men, not significant in women	Men with “<4 times/week” intake of total cruciferous vegetables had 81.31 average LINE-1 methylation% and men with “≥4 times/week” intake of total cruciferous vegetables had 82.2 average LINE-1 methylation%

Duggan et al., 2014 [24]	LINE-1	Pyrosequencing	300 overweight women aged 50–75 in the US	No differences	

Hou et al., 2010 [27]	LINE-1	Pyrosequencing	421 individuals aged 21–79 in Warsaw, Poland	No differences	

Karami et al., 2015 [29]	LINE-1	Pyrosequencing	PLCO - 436 controls from individuals aged 55–74 in the US, ATBC, 575 controls from individuals aged 55–69 in Finland	PLCO, No differences. ATBC, significant differences (*p* = 0.01) between <690.9 grams of vegetables per day and ≥690.6 grams of vegetables per day	<690.9 grams of vegetables per day have an average 78.64% LINE-1 methylation%, and ≥690.6 grams of vegetables per day have an average 78.90% LINE-1 methylation%

Liao et al., 2011 [30]	LINE-1	Pyrosequencing	654 individuals aged 20–79 from the Central and Eastern European Renal Cancer Study (CEERCC)	No differences	

Martín-Núñez et al., 2014 [51]	LINE-1	Pyrosequencing	155 individuals aged 40–65 from Spain	LINE-1 methylation increased in the control group (*p* = 0.001) but decreased in the Mediterranean diet intervention group (*p* = 0.003)	The control group had 66.8 average LINE-1 methylation% and the intervention group had 63.6 average LINE-1 methylation% after one year. Adjusted for age, gender, BMI at baseline

Tajuddin et al., 2013 [47]	LINE-1	Pyrosequencing	892 individuals aged 20–81 from the Spanish Bladder Cancer/EPICURO study, Spain	No differences	Adjusted for age, sex, region, smoking status

Zhang et al., 2012 [37]	LINE-1	Pyrosequencing	165 individuals aged 18–78 from the COMIR (Commuting Mode and Inflammatory Response) study, NY	No differences	

Fruit					

Agodi et al., 2015 [21]	LINE-1	Pyrosequencing	177 women aged 13–50, Helsinki	Significant differences (*p* = 0.022) between fruit intake groups of <201 grams/day and <201 grams/day	Data given in tertiles of methylation; women with <201 grams/day fruit intake had lower average LINE-1 methylation% than women with >201 grams/day fruit intake

Hou et al., 2010 [27]	LINE-1	Pyrosequencing	421 individuals aged 21–79 in Warsaw, Poland	No differences	

Karami et al., 2015 [29]	LINE-1	Pyrosequencing	PLCO, 436 controls from individuals aged 55–74 in the US, ATBC, 575 controls from individuals aged 55–69 in Finland	PLCO, No differences. ATBC, No differences	

Tajuddin et al., 2013 [47]	LINE-1	Pyrosequencing	892 individuals aged 20–81 from the Spanish Bladder Cancer/EPICURO study, Spain	No differences	Adjusted for age, sex, region, smoking status

Zhang et al., 2012 [37]	LINE-1	Pyrosequencing	165 individuals aged 18–78 from the COMIR (Commuting Mode and Inflammatory Response) study, NY	No differences	

Folate					

Agodi et al., 2015 [21]	LINE-1	Pyrosequencing	177 women aged 13–50, Helsinki	Significant differences (*p* = 0.027) between folate deficient and non-folate deficient groups	Data given in tertiles of methylation; women with folate deficiency had lower average LINE-1 methylation% than women without folate deficiency

Bae et al., 2014 [52]	LINE-1	LC-MS/MS	408 women aged 50–79 from the WHI-OS cohort, throughout the US	Significant differences (*p* = 0.05) among different levels of RBC folate	Women in “highest RBC folate group” had 5.12 baseline LINE-1 methylation% and women in “lowest RBC folate group” had 4.99 baseline LINE-1 methylation%

Gomes et al., 2012 [26]	LINE-1	ELISA	126 individuals aged 60–88, Brazil	No differences	

Hou et al., 2010 [27]	LINE-1	Pyrosequencing	421 individuals aged 21–79 in Warsaw, Poland	No differences	

Hsiung et al., 2007 [28]	LINE-1	COBRA PCR	765 individuals aged 18–75, Greater Boston Metropolitan Area	No differences	Adjusted for age, sex, race, smoking, alcohol, HPV serology, MTHFR

Karami et al., 2015 [29]	LINE-1	Pyrosequencing	PLCO, 436 controls from individuals aged 55–74 in the US, ATBC, 575 controls from individuals aged 55–69 in Finland	PLCO, No differences. ATBC, No differences	

Perng et al., 2014 [34]	LINE-1	Pyrosequencing	987 adults aged 45–84 from the Multi-Ethnic Study of Atherosclerosis (MESA), NY & LA	No differences	

Tajuddin et al., 2013 [47]	LINE-1	Pyrosequencing	892 individuals aged 20–81 from the Spanish Bladder Cancer/EPICURO study, Spain	No differences	Adjusted for age, sex, region

Xu et al., 2012 [11]	LINE-1	Pyrosequencing	1101 women aged 20–98, from The Long Island Breast Cancer Study Project	No differences	

Zhang et al., 2011 [36]	LINE-1	Pyrosequencing	161 individuals aged 45–75 from the North Texas Healthy Heart Study	No differences	

Zhang et al., 2012 [37]	LINE-1	Pyrosequencing	165 individuals aged 18–78 from the COMIR (Commuting Mode and Inflammatory Response) study, NY	Significant differences (*p* = 0.007) among different levels of dietary folate from fortified foods	Dietary folate from fortified foods, *µ*g/1,000 kJ spearman value 0.21

Moore et al., 2008 [44]	5-mdC	HPCE, HpaII digest, densitometry	397 individuals aged 20–81 from the Spanish Bladder Cancer Study, Spain	No differences	

Menopause status					

Xu et al., 2012 [11]	LINE-1	Pyrosequencing	1101 women aged 20–98, from The Long Island Breast Cancer Study Project	No differences	

Choi et al., 2009 [1]	5-mdC	LC/ESI-MS/MS	180 women aged 35–75	No differences	

Fetal Birthweight					

Michels et al., 2011 [53]	LINE-1	Pyrosequencing	319 mother-child dyads from Brigham and Women's Hospital, Boston	Significant differences between low birthweight (*p* = 0.007) and high birthweight (*p* = 0.036) compared to normal birthweight infants	“Low birthweight, <2500 g” had a −0.82 change in LINE-1 methylation% and “High birthweight, 4000+ g” had a −0.43 change in LINE-1 methylation%

Family history of breast cancer					

Brennan et al., 2012 [9]	LINE-1	Pyrosequencing	769 individuals aged 23–83 from 3 cohorts, USA	No differences	

Delgado-Cruzata et al., 2014 [54]	LINE-1	MethyLight	333 unaffected women who had a sister with breast cancer from the Breast Cancer Family Registry, NY	No differences	

Wu et al., 2011 [55]	LINE-1	Pyrosequencing, MethyLight	51 girls aged 6–17, USA	No differences	

Xu et al., 2012 [11]	LINE-1	Pyrosequencing	1101 women aged 20–98, from The Long Island Breast Cancer Study Project	No differences	

Wu et al., 2011 [55]	Alu	MethyLight	51 girls aged 6–17, USA	Significant differences (*p* < 0.05) between family history and no family history	Family history had 151.4 average Alu methylation% while no family history had 169.8 average Alu methylation%

Choi et al., 2009 [1]	5-mdC	LC/ESI-MS/MS	180 women aged 35–75	No differences	

Age at Menarche					

Choi et al., 2009 [1]	5-mdC	LC/ESI-MS/MS	180 women aged 35–75	No differences	

Age at first birth					

Choi et al., 2009 [1]	5-mdC	LC/ESI-MS/MS	180 women aged 35–75	No differences	

Parity					

Choi et al., 2009 [1]	5-mdC	LC/ESI-MS/MS	180 women aged 35–75	No differences	

Hormone Cycle					

El-Maarri et al., 2011 [25]	LINE-1	Pyrosequencing, SIRPH	500 individuals aged 18–64, Bonn, Germany	No differences	

Sex Hormones					

Iwasaki et al., 2012 [56]	LINE-1	LUMA	185 women aged 55–74, Japan	No differences	

Ulrich et al., 2012 [57]	LINE-1	Pyrosequencing	173 women aged 55–75 from the Physical Activity for Total Health Study	No differences	

Hormone use					

Choi et al., 2009 [1]	5-mdC	LC/ESI-MS/MS	180 women aged 35–75	No differences	
